# Elevated SASP Factors, Reduced Antioxidant Enzymes, and Increased Tumor Susceptibility in Space Radiation-Exposed *Apc*^Min/+^ Mice

**DOI:** 10.3390/ijms27010211

**Published:** 2025-12-24

**Authors:** Kamendra Kumar, Jerry Angdisen, Albert J. Fornace, Shubhankar Suman

**Affiliations:** 1 Department of Oncology, Lombardi Comprehensive Cancer Center, Georgetown University Medical Center, Washington, DC 20057, USA; kk1264@georgetown.edu (K.K.); jja87@georgetown.edu (J.A.);; 2 Department of Biochemistry and Molecular & Cellular Biology, Georgetown University Medical Center, Washington, DC 20057, USA

**Keywords:** space radiation, galactic cosmic radiation, cancer risk, antioxidant enzymes, senescence-associated secretory phenotype (SASP) factors, risk biomarkers

## Abstract

Human missions into deep space will expose astronauts to the unique and complex radiation environment of galactic cosmic radiation (GCR), a mixed field of high-energy protons and heavy ions predicted to substantially increase long-term cancer risk. To support effective risk stratification, early detection, and mitigation strategies, there is a need to identify biomarkers indicative of GCR-induced cancer risk. Here, we applied a Tandem Mass Tag (TMT)-based quantitative proteomics approach to identify potential biomarkers associated with GCR-induced gastrointestinal (GI) and mammary tumorigenesis using the female *Apc*^Min/+^ mouse, a well-established model of human colorectal and breast cancer. Eight- to ten-week-old *Apc*^Min/+^ mice were exposed to 75 cGy of simulated GCR and serum and tissue samples were collected 100–110 days post-exposure for molecular and histopathological analyses. Tumor incidence was scored by blinded observers, and serum proteomes exhibiting a fold change > 1.2 or <0.83 with *p* < 0.05 were considered significantly altered. Bioinformatics analyses, including Gene Ontology, Kyoto Encyclopedia of Genes and Genomes pathway enrichment, and unsupervised clustering, were employed to delineate GCR-responsive molecular networks. Validation of differentially expressed proteins (DEPs) was performed using immunoblotting, ELISA, and enzyme activity assays. GCR exposure resulted in a significant increase in both GI and mammary tumor burden relative to controls. Proteomic profiling revealed 194 upregulated and 461 downregulated proteins, distinguishing GCR-exposed from control serum proteomes. Functional enrichment analyses highlighted alterations in metabolic processes, PI3K-AKT, HIF-1, and PPAR signaling pathways, alongside the suppression of antioxidant defense mechanisms. Notably, mice exposed to GCR exhibited elevated serum levels of TGF-β1 and MMP9, accompanied by reduced levels and enzymatic activities of key antioxidant defenses. Cross-referencing 36 GCR-induced serum SASP factors with the Human Protein Atlas revealed 11 SASP proteins associated with human breast and colorectal cancers. Together, these findings show that GCR exposure triggers a pro-tumorigenic serum proteomic signature that may serve as a biomarker for assessing cancer risk in astronauts during deep-space missions.

## 1. Introduction

Manned missions beyond Earth’s magnetosphere, including future expeditions to Mars, will inevitably expose astronauts to the ionizing radiation (IR) environment of deep space [1,2,3]. The deep-space radiation environment is dominated by two principal components: galactic cosmic radiation (GCR) and solar particle events (SPEs). While SPEs consist of intermittent bursts of low- to medium-energy protons emitted from the Sun, GCR constitutes a continuous flux of high-energy charged particles, primarily protons (~85%), helium nuclei (~14%), and a small (~1%) but biologically significant fraction of high-charge, high-energy (HZE) ions [4,5,6]. Unlike low-energy protons, HZE particles can readily penetrate conventional spacecraft shielding, making GCR the dominant long-term radiation hazard for astronauts in deep space [7,8,9]. Astronauts traveling in deep space are expected to receive a cumulative absorbed dose of approximately 30–60 centigray (cGy) over the course of a round-trip mission to Mars [10,11,12,13]. This exposure is hundreds of times greater than the average annual background radiation dose on Earth, which is roughly 0.2–0.3 cGy per year [14]. Consequently, an increased health risk is anticipated for astronauts during interplanetary missions, further compounded by the presence of HZE ions, which possess high linear energy transfer (LET) properties and produce complex, densely ionizing tracks that cause severe and less-repairable biological damage [15]. Several in vivo studies have demonstrated that high-LET heavy ions (or HZE ions) present in GCR exhibit a substantially greater relative biological effectiveness (RBE) in inducing carcinogenic effects compared with both low-Z (atomic number) charged particles such as protons and low-LET photon radiation (such as X-rays or γ-rays) [16,17,18,19,20,21]. Therefore, exposure to GCR is predicted to significantly increase cancer risk during and after prolonged deep-space missions [22,23,24,25,26]. Despite growing evidence that exposure to GCR increases cancer risk across multiple tissues [22,27,28], our understanding of the systemic molecular mechanisms underlying this process remains limited [29]. In this study, we seek to bridge this knowledge gap by identifying systemic proteomic signatures of GCR exposure that can inform cancer risk assessment, guide countermeasure development, and improve astronaut health protection during deep-space missions.

Because no human long-term missions have yet ventured beyond Earth’s magnetosphere, direct evidence of GCR-induced tissue-specific cancer risk in astronauts remains unavailable. However, insights from epidemiological studies of terrestrial IR exposure including atomic bomb survivors and occupationally exposed populations have consistently demonstrated an elevated risk of malignancies in rapidly proliferating tissues, such as the gastrointestinal (GI) tract and mammary glands [30,31,32,33]. To overcome the limitations of human data, ground-based simulation experiments employing animal models and accelerator-generated GCR beams have become indispensable [5,34]. Among the available in vivo models of IR-induced cancer, mice carrying mutations in the adenomatous polyposis coli (*Apc*) gene are widely used to study both GI and mammary tumorigenesis. Notably, the female *Apc*^Min/+^ mouse harboring a germline mutation in one allele of the *Apc* tumor suppressor gene is particularly valuable because it closely recapitulates molecular features of colorectal (CRC) and breast cancer in humans [21,35,36,37,38]. Previous studies using *Apc*^Min/+^ mice have provided substantial evidence that exposure to space-relevant radiation types significantly increases the incidence of both GI and mammary tumors [17,18,21,39]. Building on these findings, the present study investigates the impact of simulated GCR exposure on the serum proteome of *Apc*^Min/+^ mice, with a particular focus on the modulation of pro-oncogenic and inflammatory senescence-associated secretory phenotype (SASP) factors, as well as the dysregulation of antioxidant defense mechanisms involved in cancer suppression [40,41]. The SASP factors are a complex mixture of pro-inflammatory cytokines, chemokines, growth factors, and matrix metalloproteinases (MMPs). While both IR- and oncogene-induced SASP are known to play a context-dependent role in tumor development, persistent systemic accumulations of SASP factor have been implicated in accelerating neoplastic transformation by enhancing tissue inflammation, angiogenesis, and extracellular matrix remodeling [42,43,44,45]. Conversely, antioxidant enzymes, including superoxide dismutase (SOD), glutathione peroxidase (GPX), and catalase (CAT), serve as frontline defenses against reactive oxygen species (ROS), such as those produced by pro-inflammatory signaling. Dysregulation or depletion of these enzymes disrupts redox homeostasis, exacerbating oxidative stress and promoting mutagenic and pro-tumorigenic signaling cascades [46,47,48].

Using TMT-based quantitative proteomics coupled with bioinformatics analyses, including Gene Ontology (GO) classification, Kyoto Encyclopedia of Genes and Genomes (KEGG) pathway mapping, and cluster analysis, we comprehensively profiled the serum proteome alterations in GCR-exposed *Apc*^Min/+^ mice. Together, these findings suggest that exposure to simulated GCR induces a pro-tumorigenic systemic proteomic signature in *Apc*^Min/+^ mice, characterized by elevated SASP factors and reduced antioxidant enzyme activity in serum, which coincides with increased tumor susceptibility. This molecular profile provides new mechanistic insights into space radiation-induced carcinogenesis and identifies serum-based biomarker candidates with potential applications in cancer risk assessment, radiation countermeasure development, and the protection of astronaut health during future deep-space missions.

## 2. Results

### 2.1. GCR Exposure Promotes GI and Mammary Tumorigenesis in Apc^Min/+^ Mice

**Figure 1:**
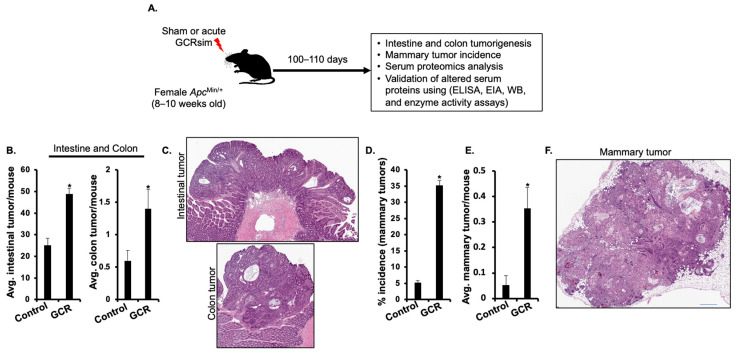
GCR-induced gastrointestinal and mammary tumorigenesis in female *Apc*^Min/+^ mice. (**A**) Schematic summary of experimental design. (**B**) Mean (± SEM) intestinal and colon tumor numbers in control (*n* = 15) and GCR exposed mice (*n* = 15). (**C**) Representative micrograph of H&E stained intestinal and colonic tumors. (**D**) Mammary tumor frequency in control (*n* = 38) and GCR exposed mice (*n* = 34). (**E**) Mean (±SEM) mammary tumor numbers in control (*n* = 38) and GCR exposed mice (*n* = 34). (**F**) Representative micrograph of H&E-stained mammary gland tumor. * Depicts statistically significant change relative to the control group. Scale 250 μm.

Female *Apc*^Min/+^ mice exposed to 75 cGy acute GCR exhibited significantly increased GI and mammary tumorigenesis compared to controls. Mean intestinal tumor burden was approximately twofold higher in GCR-irradiated mice (48.9 ± 2.5) compared with controls (25.1 ± 3.3; *p* < 0.05). Similarly, colon tumor multiplicity was significantly increased following GCR exposure (1.4 ± 0.3) relative to control mice (0.6 ± 0.16; *p* < 0.05). (Figure 1A–C). In the mammary gland, GCR exposure increased tumor incidence from 5.3 ± 0.6% in controls to 35.3 ± 1.4% (*p* < 0.05) (Figure 1D). Mean mammary tumor frequency was significantly increased in GCR-irradiated mice compared with controls (control: 0.05 ± 0.04; GCR: 0.35 ± 0.08; *p* < 0.05; Figure 1E,F). These data indicate that GCR exposure enhances both GI and mammary tumor development in female *Apc*^Min/+^ mice.

### 2.2. GCR Exposure Alters the Serum Proteome in Apc^Min/+^ Mice

**Figure 2:**
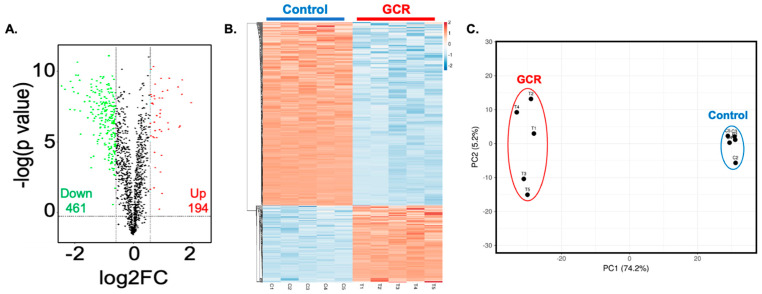
Identification of and characterization of differentially expressed proteins (DEPs). (**A**) Volcano plot of DEPs. This plot depicts log_2_ fold-change (*x*-axis) versus -log_10_ *p* value (*y*-axis), representing the probability that the protein is differentially expressed (*n* = 5/group). *p* value ≤ 0.05, Fold change (FC) > 1.2 and <0.83 are set as the threshold to detect significant changes in protein expression. Red dots depict up-regulated and green dots depicts down-regulated proteins, and black dot indicates no significant alterations. (**B**) Cluster analysis of DEPs depicted in the form of a heatmap. (**C**) Principle component analysis (PCA) plot showing a clear separation of experimental groups based on their DEP profile demonstrating their biomarker feature.

To investigate systemic alterations associated with GCR-induced GI and mammary tumorigenesis, we performed serum proteomic profiling in control and GCR-exposed *Apc*^Min/+^ mice. A total of 655 DEPs were identified, with 194 upregulated and 461 downregulated in the GCR group (Figure 2A). Hierarchical clustering revealed clear segregation of samples based on exposure status (Figure 2B), which was further confirmed by principal component analysis (PCA) showing distinct clustering of control and GCR groups (Figure 2C).

**Figure 3:**
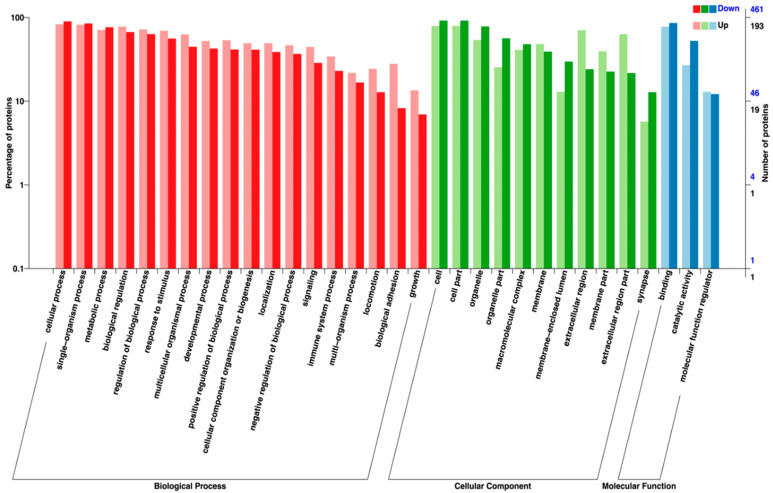
Gene ontology (GO) based distribution of DEPs in relation to their function in biological process (red), cellular component (green) and molecular function (blue). Light-shaded bars represent upregulated proteins, while dark-shaded bars represent downregulated proteins, as indicated by the color key in the upper right quadrant. The secondary vertical axis (right) provides the absolute Number of proteins down (deep blue) or up (black) associated with each GO term.

**Figure 4:**
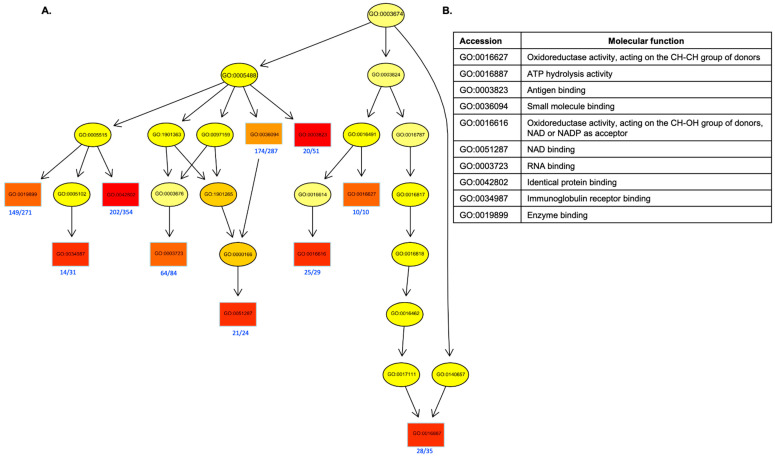
Directed acyclic graph (DAG) analysis. (**A**) Each GO term represents a specific biological concept and is organized in a hierarchical structure, resembling an upside-down tree. Boxes indicate the 10 most significant terms. Box color represents the relative significance, ranging from dark red (most significant) to light yellow (least significant). Gene Ontology (GO) accession numbers and the associated fraction of DEPs are shown in blue font. (**B**) List of 10 most significant terms GO term accession number and name of corresponding molecular function.

GO analysis indicated that DEPs were predominantly enriched in biological processes related to metabolism, stress response, and immune regulation (Figure 3). Directed acyclic graph (DAG) analysis highlighted significant enrichment in oxidoreductase activity, ATP hydrolysis activity, and antigen binding, among other molecular functions (Figure 4A,B).

**Figure 5:**
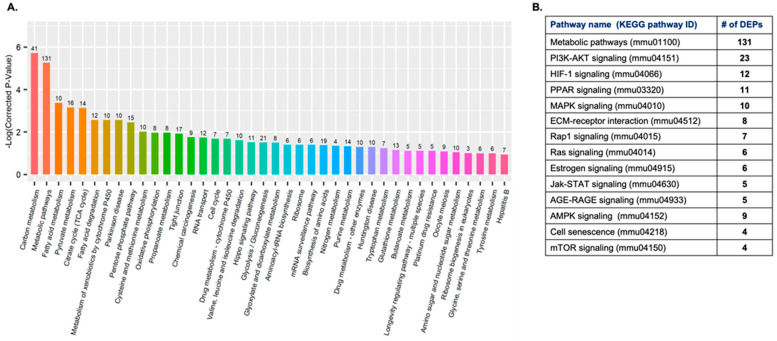
Functional annotation using KEGG pathway analysis. (**A**) KEGG enrichment (sorted by *p*-value) analysis. (**B**). A table of the top 14 altered signaling pathways with number of DEPs in each signaling network.

**Figure 6:**
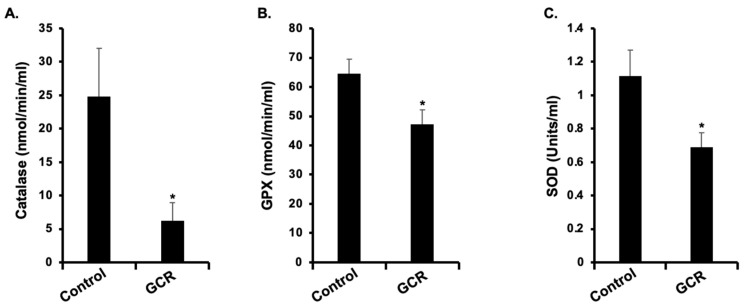
Validation of serum proteomics data using enzyme activity assay (*n* = 6/group). (**A**) Catalase enzyme activity (**B**) Glutathione peroxidase (GPX) enzyme activity (**C**) Superoxide dismutase (SOD) enzyme activity. * Depicts statistically significant change relative to the control group.

KEGG pathway analysis identified 40 significantly altered pathways, with the most enriched being metabolic pathways (131 DEPs), PI3K–Akt signaling (23 DEPs), and HIF-1 signaling (12 DEPs) (Figure 5A,B). Several cancer-relevant signaling pathways, including PPAR, MAPK, Ras, and mTOR signaling, were also significantly enriched. The top 25 upregulated and downregulated serum proteins are listed in Table 1 and Table 2. The top downregulated proteins were mainly involved in metabolic and stress response pathways, such as isocitrate dehydrogenase [NADP], elongation factor 1-alpha 2, and L-lactate dehydrogenase B chain. Furthermore, an analysis of significantly altered antioxidant enzymes/proteins showed that several enzymes were significantly downregulated in the GCR-exposed mice, as detailed in Table 3. Further, the activities of key antioxidant enzymes, i.e., catalase, SOD, and GPX, were significantly reduced in the GCR group compared with the controls. Catalase activity decreased from 24.82 ± 7.2 in the control group to 6.3 ± 2.6 in the experimental group (Figure 6A). Similarly, GPX activity was significantly lower in the experimental group (47.2 ± 6.9) compared with controls (64.5 ± 2.9; Figure 6B). SOD activity also showed a marked reduction in the experimental group (0.69 ± 0.09) relative to the control group (1.1 ± 0.2; Figure 6C). These results indicate a significant decrement in the antioxidant defense system. Together, these findings demonstrate that GCR exposure induces widespread changes in the serum proteome of *Apc*^Min/+^ mice, affecting both antioxidant enzyme system, and oncogenic signaling networks.

### 2.3. GCR Exposure Upregulates SASP-Associated Serum Proteins

**Figure 7:**
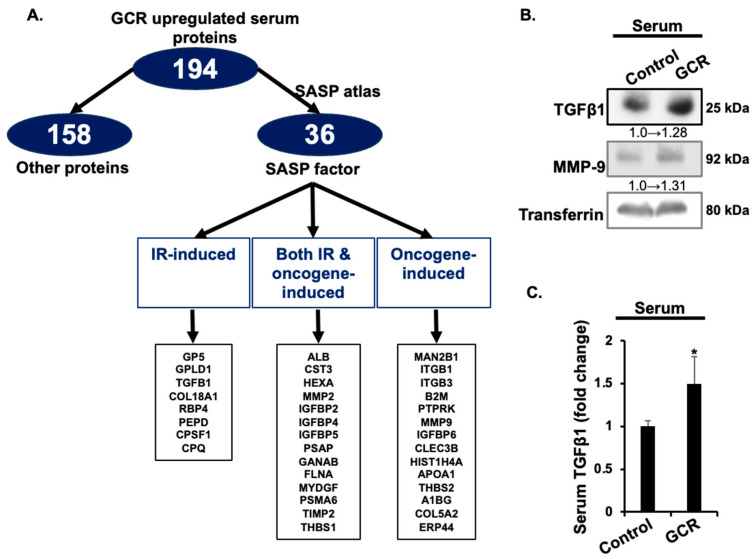
Association between GCR-induced serum proteins and IR- or oncogene-induced SASP factors. (**A**) Out of 194 upregulated serum proteins, 36 were identified as SASP-derived proteins using SASP atlas database (http://www.saspatlas.com, accessed on 25 August 2025). Classification of GCR-induced serum SASP factors as IR- or oncogene-induced SASP factors. (**B**) Validation of serum SASP factors (TGFβ1 and MMP9) using Western blotting. Normalized fold change relative to serum transferrin has been provided adjacent to the TGFb1 and MMP-9 blots. (**C**) ELISA based validation of GCR-induced increase in serum TGFβ1 level (*n* = 6/group). * Depicts statistically significant change relative to the control group.

To explore whether GCR-induced proteomic alterations overlap with senescence-associated secretory phenotype (SASP) factors, we compared the 194 upregulated serum proteins with the SASP atlas database. Thirty-six proteins were identified as SASP-associated, including those induced by IR, oncogenic stress, or both (Figure 7A).

As detailed in Table 4, key SASP factors that were upregulated included Matrix metalloproteinase 9 (MMP9), Transforming growth factor beta-1 (TGFβ1), and multiple Insulin-like growth factor-binding proteins (IGFBP2, IGFBP4, IGFBP6, IGFBP5). Other notable upregulated SASP proteins were Thrombospondin-1 (THBS1), Retinol-binding protein 4 (RBP4), and Prosaposin (PSAP). Western blot analysis confirmed increased levels of TGFβ1 and MMP9 in the serum of GCR-exposed mice compared to controls (Figure 7B). Consistent with this, ELISA assays validated a significant increase in circulating TGFβ1 following GCR exposure (1.49 ± 0.3) compared with controls (1.0 ± 0.06; Figure 7C). To validate functional consequences of the proteomic changes, enzymatic assays were performed. Collectively, these results indicate that GCR exposure enhances circulating SASP factors, consistent with systemic pro-tumorigenic and inflammatory signaling.

### 2.4. GCR-Induced SASP Factors in Apc^Min/+^ Mice Overlap with Human Cancer Proteomes

To determine the clinical relevance of the GCR-induced serum SASP factors in *Apc*^Min/+^ *mice* and its association with human cancer development, we analyzed their expression in human cancer tissues using the HPA database. We focused on breast and colon cancer tissues. Our analysis, summarized in Table 5, revealed that many of the GCR-induced 11 SASP proteins are also highly expressed in human breast and colon cancer tissues. For instance, Prosaposin (PSAP), Endoplasmic reticulum resident protein 44 (ERP44), Proteasome subunit alpha type-6 (PSMA6), and Histone H4 (HIS1H4A) were highly expressed in most of the breast and colorectal cancer samples. Other proteins showed varying expression patterns. Cystatin-C (CST3) and Xaa-Pro dipeptidase (PEPD) were highly expressed in colon cancer samples compared to breast cancer. Thrombospondin-1 (THBS1) and Metalloproteinase inhibitor 2 (TIMP2) were also frequently expressed in both tissue types. In contrast, Insulin-like growth factor-binding protein 6 (IGFBP6) has low frequency in both colon and breast cancers. These findings indicate a significant overlap between the circulating SASP signature induced by GCR exposure in mice and the proteomic profiles of human breast and CRC tumors, suggesting that GCR-induced systemic SASP factors may contribute to tumor development in these human cancers.

## 3. Discussion

This study demonstrates integrated in vivo phenotypic and systemic proteomic evidence that exposure to GCR significantly enhances the risk of GI and mammary tumor development, accompanied by widespread alterations in the serum proteome, including the upregulation of SASP factors and downregulation of antioxidant enzymes. These results collectively reveal that space radiation exposure may accelerate tumor development through redox imbalance and chronic accumulation of pro-tumorigenic SASP factors. The increased intestinal tumor burden and the pronounced increase in mammary tumor incidence observed in GCR-exposed *Apc*^Min/+^ mice are consistent with a growing body of evidence demonstrating the potent oncogenic potential of space radiation [17,27,49,50,51,52]. Previous studies have shown that exposure to individual HZE ions, such as ^56^Fe or ^28^Si, markedly accelerates intestinal tumorigenesis in animal models compared with γ-ray exposure [17,19,24]. Proton irradiation, although lower in LET but significant proportion of GCR, has also been reported to exacerbate both intestinal neoplasia and mammary tumor incidence in *Apc*^Min/+^ mice [17,18,20]. More recently, mixed-field GCR studies have also revealed enhanced tumorigenesis accompanied with distinct molecular alterations, including SASP signaling driven oncogenic and inflammatory pathway activation [19,21,27,53]. Collectively, these findings highlight that multi-ion GCR-like exposures elicit broader biologically consequential effects and represents a critical oncogenic risk for astronauts exposed to deep-space radiation environments.

Our serum proteomics analysis revealed extensive molecular reprogramming at the systemic level following GCR exposure, characterized by the downregulation of key antioxidant enzymes, indicative of a compromised oxidative defense system. This observation aligns with findings from both human and animal studies demonstrating long-term suppression of enzymatic antioxidants after IR exposure. Epidemiological studies of occupationally and accidentally exposed populations, including Chernobyl recovery workers, have consistently reported reduced activities of SOD, CAT, and GPX, along with biochemical evidence of chronic oxidative stress [54,55]. Similarly, healthcare workers exposed to diagnostic X-rays exhibit significantly decreased erythrocyte SOD, CAT, and GPX activities compared with unexposed controls, supporting persistent oxidative imbalance in humans chronically exposed to low-dose radiation [56,57,58,59]. In animal models, IR exposure results in a LET-dependent sustained decline in antioxidant enzyme activity across multiple tissues, with GPX, CAT, and SOD levels remaining depressed months after exposure [46,60,61]. Studies further demonstrate that pharmacological restoration of these antioxidant enzymes using mimetics agents mitigates radiation-induced late tissue injury, underscoring the causal role of compromised antioxidant defenses in chronic radiation pathology [62,63]. This persistent redox imbalance may exacerbate DNA and lipid oxidation, mitochondrial dysfunction, and redox-sensitive signaling alterations, such as activation of PI3K-AKT and MAPK pathways, all of which are hallmarks of IR-induced cellular stress and carcinogenic transformation [64,65,66,67]. Supporting convergence on metabolic and growth-promoting pathways include our KEGG and GO enrichment analyses of proteomic data implicated altered metabolic, PI3K-AKT and mTOR signaling pathways. These findings are consistent with previous multi-omics studies demonstrating that GCR or other high-LET radiation suppresses antioxidant networks, disrupts metabolism, and activates redox-sensitive signaling cascades such as PI3K-AKT, MAPK, and mTOR [54,68,69,70].

The finding that GCR exposure accelerates tumorigenesis is further supported by evidence that the antioxidant system declines in an accelerated aging-like manner. The simultaneous suppression of antioxidant enzymes and induction of SASP factors points to a direct and often bidirectional crosstalk between oxidative stress and senescence signaling. Specifically, oxidative stress activates the DNA Damage Response, which, through subsequent cell cycle checkpoint activation, enforces the state of cellular senescence. Conversely, the resulting senescent cells secrete various SASP factors that then further amplify oxidative and inflammatory processes in the microenvironment. This cyclic interaction establishes a self-reinforcing loop that effectively sustains redox imbalance and pro-tumorigenic signaling long after the initial molecular insult has ceased [71,72,73,74]. Space-relevant radiation types including GCR are known to produce persistent oxidative stress, chronic DNA damage signaling, and premature senescence in intestinal and other stem/progenitor compartments phenotypes. Prior studies have also shown that IR, especially high-LET radiation induces a robust SASP characterized by increased secretion of TGFβ1, MMP9, IL-6, and IGFBPs, promoting chronic inflammation and tumor progression [15,75,76,77]. Moreover, senolytic-based interventions have also demonstrated ability to mitigate SASP accumulations and GI tumor development after exposure to both low- and high-LET IR [45]. Multiple SASP-linked proteins that rose in GCR-exposed mouse serum including, PSAP, THBS1, TIMP2, CST3, and PSMA6 are broadly detected in human malignancies, including breast and colorectal cancers were upregulated after GCR exposure [78,79,80,81,82,83]. Moreover, ERP44 and histone H4 family members are reported to be broadly expressed across multiple tumor types [84,85], supporting their potential relevance as cross-tissue markers. However, it should be noted that expression frequency and functional context vary considerably by cancer type, and the present cross-referencing analysis does not establish a causal relationship between GCR exposure and tumorigenesis. Rather, the observed aggregate pattern suggests a putative conserved, cancer-associated signature that partially overlaps with the GCR-induced SASP.

The induction of a systemic SASP-like secretory phenotype, together with the suppression of antioxidant defense networks, indicates that even sublethal doses of GCR can reprogram tissue homeostasis toward a persistently pro-tumorigenic state. While these coordinated molecular changes are consistent with pro-tumorigenic signaling pathways, their direct role in cancer initiation or progression remains speculative and warrants further functional validation. Consequently, these molecular alterations should be interpreted as candidate biomarkers for early risk assessment and for monitoring the efficacy of radioprotective or chemopreventive interventions, rather than definitive predictors of cancer development.

Furthermore, the partial overlap between GCR-induced serum proteins and human cancer-associated proteomes suggests that space radiation exposure may recapitulate molecular features linked to aging, chronic inflammation, and sporadic carcinogenesis on Earth. However, these observations were derived from the tumor-prone *Apc*^Min/+^ model, and baseline proteomic differences between *Apc*^Min/+^ and wild-type mice, particularly the absence of spontaneous gastrointestinal tumorigenesis in wild-type animals, represent an important limitation for direct translational interpretation. Accordingly, future investigations that compare GCR-induced proteomic responses across *Apc*^Min/+^ and wild-type backgrounds, and integrate these findings with data from humanized experimental systems and well-characterized astronaut cohorts, will be essential for validating broadly applicable biomarkers. Such efforts will not only advance space radiation biomarker discovery and therapeutic development but also improve our understanding of shared molecular mechanisms underlying stress-induced malignancy and aging-related pathologies in humans.

## 4. Materials and Methods

### 4.1. Animal Breeding, Irradiation, and Maintenance

Male *Apc*^Min/+^ mice (JAX stock# 002020, The Jackson Laboratory, Bar Harbor, ME, USA) on a C57BL/6J background were bred with female C57BL/6J mice (JAX stock# 000664) in the Georgetown University (GU) Animal Research Facility. Offspring were genotyped to identify *Apc*^Min/+^ heterozygous mice following the standard genotyping protocol provided by The Jackson Laboratory (https://www.jax.org/Protocol?stockNumber=002020&protocolID=529; accessed on 1 October 2015). Following genotyping, female *Apc*^Min/+^ mice were assigned to experimental groups and transported to the Brookhaven National Laboratory (BNL, Upton, NY, USA) animal facility using an approved laboratory animal courier service. Upon arrival, mice were acclimatized for one week prior to irradiation. At the age of approximately 10 weeks, animals were either sham-irradiated or exposed to 75 cGy (~0.3 to 0.4 cGy/min averaged dose rate including time of beam switching) of simulated GCR (referred as GCR hereafter) at the NASA Space Radiation Laboratory (NSRL). The acute full spectrum (33-ion) GCR exposure comprised seven different ion species, i.e., hydrogen (H), helium (He), carbon (C), oxygen (O), silicon (Si), titanium (Ti), and iron (Fe) ions, across an energy range of 20–1000 MeV/nucleon. This beam composition mimics the charge and energy distribution of the galactic cosmic radiation field experienced beyond Earth’s magnetosphere [5,34]. Following irradiation, all animals including control and irradiation groups were shipped back on the next available day to the GU animal facility and were maintained until completion of the study. The study was conducted in two independent beam runs. In the initial beam run, 15 mice per group were sufficient to achieve statistically significant outcomes for GI tumorigenesis, whereas larger cohort sizes required to reach statistical significance for mammary tumorigenesis was added in the subsequent beam run. The number of mice analyzed per group is provided in the corresponding figure legends.

At both GU and BNL animal facilities, animals were group-housed (five per cage) in specific pathogen-free (SPF) conditions within individually ventilated cages containing autoclaved bedding. The animal rooms were maintained at 22 ± 2 °C and 50 ± 10% relative humidity, under a 12-h light–dark cycle. Mice were provided a standard rodent diet and filtered water ad libitum. Animals were monitored daily for signs of distress, including hunched posture, ruffled fur, diarrhea, decreased activity, or >15% body weight loss. Any mouse exhibiting severe distress or declining health was humanely euthanized by carbon dioxide (CO_2_) asphyxiation and excluded from further analyses. During the course of the study, two mice in the control group and one mouse in the GCR-exposed group were euthanized due to severe distress and health decline that met predefined euthanasia criteria and were excluded from subsequent analyses. All experimental procedures were conducted in accordance with the Guide for the Care and Use of Laboratory Animals (Institute of Laboratory Animal Resources, National Research Council, U.S. National Academy of Sciences) and approved by the Institutional Animal Care and Use Committee (IACUC) at Georgetown University (Protocol #2019-0070; initial approval date: 11 March 2020) and at Brookhaven National Laboratory (Protocol #515; initial approval date: 7 January 2020).

### 4.2. Euthanasia and Serum Collection

At 100–110 days post-irradiation, all animals were humanely euthanized using a carbon dioxide (CO_2_) chamber with a regulated flow rate of 30–60% of the chamber volume per minute, in compliance with approved IACUC protocol. This 100–110 day timepoint was selected based on our prior studies in *Apc*^Min/+^ mice, which demonstrated that this interval provides an optimal window to detect radiation-induced GI and mammary tumorigenesis while minimizing confounding attrition resulting from the model’s propensity for spontaneous tumor development [17,18,20,39].

Immediately following euthanasia, whole blood was collected via cardiac puncture using sterile syringes and transferred into serum separator tubes (BD Microtainer^®^ Ref. No. 365967; Becton, Dickinson and Company, Franklin Lakes, NJ, USA). Blood samples were allowed to clot at room temperature and then centrifuged according to the manufacturer’s protocol to isolate serum. The resulting serum was aliquoted, flash-frozen in liquid nitrogen, and stored at −80 °C until subsequent proteomic and biochemical analyses. An overview of the experimental plan, biospecimen collection, and subsequent molecular and histological analyses are summarized in Figure 1A. Serum samples used for proteomic analyses were collected exclusively during the first beam run.

### 4.3. Quantitative Analysis of GCR-Induced Gastrointestinal and Mammary Tumorigenesis

Following euthanasia and blood collection, GCR-exposed and age-matched sham-irradiated control mice were processed in parallel for mammary and GI tumor quantitation. To quantify mammary tumors, the mammary fat pads were surgically exposed, and macroscopic mammary tumors were visually identified and quantified by two blinded observers. To quantify GI tumors, the small intestine and colon tissues were excised, flushed with phosphate-buffered saline to remove luminal contents, placed on absorbent paper, and longitudinally opened. Visible GI tumors throughout the intestinal and colon tissue were counted under a dissecting microscope (Leica MZ6, Leica Microsystems, Buffalo Grove, IL, USA) by independent observers who were blinded to the experimental groups. After quantification, mammary and GI tumors were then fixed in 10% buffered formalin for 24 h, then transferred to 70% ethanol prior to paraffin embedding. Formalin-fixed, paraffin-embedded (FFPE) sections of ~5 μm thickness were deparaffinized in xylene, rehydrated through graded ethanol, and stained with hematoxylin to visualize nuclei, followed by eosin for cytoplasmic counterstaining. Stained slides were finally imaged using an Aperio whole-slide scanner (Leica Biosystems, Buffalo Grove, IL, USA).

### 4.4. Serum Proteomics Analysis

In order to deplete highly abundant proteins, serum samples were added directly to the resin slurry, incubated with gentle mixing for 30 min at room temperature, and centrifuged at 1000× *g* for 2 min. The flow-through containing albumin- and immunoglobulin G (IgG)-depleted serum proteins was collected for downstream analysis. A total of 100 μg of protein from each sample was reduced with 10 mM TCEP [tris(2-carboxyethyl)phosphine] at 56 °C for 1 h, followed by alkylation with 20 mM iodoacetamide (IAA) at room temperature in the dark for 1 h. Samples were digested overnight at 37 °C with sequencing-grade trypsin (enzyme-to-substrate ratio 1:50). Resulting peptides were lyophilized to near dryness and reconstituted in 100 mM triethylammonium bicarbonate (TEAB). Peptides were labeled with tandem mass tag (TMT) 10-plex reagents (Thermo Fisher Scientific, Waltham, MA, USA) according to the manufacturer’s instructions. Briefly, TMT reagents were dissolved in 20 μL anhydrous acetonitrile (ACN), added to peptide solutions, and incubated for 1 h at room temperature. Labeling reactions were quenched with 8 μL of 5% hydroxylamine for 15 min. Labeled samples were combined in equal amounts and pooled for analysis. The pooled TMT-labeled peptide mixtures were fractionated into 12 components using high-pH reverse-phase high-performance liquid chromatography (HPLC). Samples were then analyzed on an Ultimate 3000 nano-ultra-high-performance liquid chromatography (nano-UHPLC) system (Thermo Fisher Scientific) coupled to a Q Exactive HF mass spectrometer (Thermo Fisher Scientific) with a Nanospray Flex Ion Source. Peptides were separated on a PepMap C18 trapping column (100 Å, 100 μm × 2 cm, 5 μm) and an analytical column (100 Å, 75 μm × 50 cm, 2 μm) at a flow rate of 250 nL/min. Mobile phases consisted of 0.1% formic acid (FA) in water (A) and 0.1% FA in 80% ACN (B). The gradient was 5–7% B in 2 min, 7–20% B in 20 min, 20–40% B in 34 min, and 40–90% B in 4 min. Mass spectrometry (MS) data were acquired in data-dependent Top15 mode with a full MS scan range of 350–1650 *m*/*z* at 120,000 resolution [automatic gain control (AGC) target: 3 × 10^6^]. MS/MS scans were acquired at 30,000 resolution (AGC target: 1 × 10^5^), with normalized collision energy set to 32%, an isolation window of 1.2 Th, dynamic exclusion of 40 s, and exclusion of unassigned, +1, and >+6 charge states. Raw MS files were processed using MaxQuant v1.6.2.14 against the UniProt *Mus musculus* database. Search parameters included: fixed modification of carbamidomethylation (C), variable modification of oxidation (M), TMT-10plex labeling, trypsin specificity with up to 2 missed cleavages, precursor ion mass tolerance of 10 ppm, and MS/MS tolerance of 0.6 Da.

A total of 1374 proteins were identified. Proteins with fold-change (FC) ≥ 1.2 were considered upregulated, while those with FC ≤ 0.83 (1/1.2) were considered downregulated. This threshold was selected based on the study’s objective to characterize long term systemic effects following exposure to a modest 75 cGy dose of GCR, a dose chosen to model the protracted, low-level exposures encountered during space travel. Such a modest GCR dose is expected to elicit widespread but subtle alterations in the circulating proteome, rather than the large, acute shifts typical of high-dose irradiation. Notably, the top 25 most upregulated and top 25 most downregulated proteins (Table 1 and Table 2) apply substantially more stringent fold-change thresholds (minimum log_2_FC of 0.80 for upregulated proteins and maximum log_2_FC of −1.41 for downregulated proteins), ensuring that the most robust and biologically relevant proteomic changes are highlighted. The full list of identified proteins and DEPs is provided in the Supplementary Material.

### 4.5. Western Blotting

Serum samples were prepared for immunoblotting to validate the proteomic findings. Equal volumes of mouse serum were diluted 1:1 in a RIPA buffer (Thermo Scientific, Pierce, Rockford, IL, USA) containing protease and phosphatase inhibitors (Boston Bioproducts, Milford, MA, USA), and then 4X reducing Laemmli buffer (Thermo Scientific) was added with a final concentration of 1X. The samples were then denatured by heating at 95 °C for 10 min. Proteins were separated by SDS-PAGE using precast 4–20% Tris-Glycine gels (Bio-Rad, Hercules, CA, USA). The resolved proteins were then transferred to a PVDF membrane (Thermo Scientific, Pierce). Following the transfer, the membranes were blocked with 5% non-fat milk in Tris-buffered saline with 0.1% Tween 20 (TBST) for 1 h at room temperature to prevent non-specific antibody binding. The membranes were then incubated overnight at 4 °C with the following primary antibodies: anti-TGF1 (1:1000 dilution; catalog No. sc-31609, Santa Cruz Biotechnology, Dallas, TX, USA), anti-MMP9 (1:1000 dilution; catalog No. ab38898, Abcam, Waltham, MA, USA), and anti-Transferrin (1:1000 dilution; catalog No. PA5-27306, Thermo Scientific). Transferrin was used as a loading control to normalize protein levels across samples. After primary antibody incubation, the membranes were washed three times for 10 min each with TBST. They were then incubated for 1 h at room temperature with the appropriate horseradish peroxidase (HRP)-conjugated secondary antibodies (Cell Signaling Technology, Danvers, MA, USA) diluted 1:2500 in TBST. The membranes were washed again three times for 10 min each with TBST. Protein bands were visualized using the SuperSignal™ West Pico PLUS Chemiluminescent Substrate (Thermo Scientific) and a ChemiDoc™ Imaging System (GE Healthcare, Chicago, IL, USA). Densitometric analysis of the bands was performed using Fiji (ImageJ2, version 2.15.1/ImageJ 1.54f) software (NIH, Bethesda, MD, USA) to quantify protein expression, with values normalized to the Transferrin loading control [86].

### 4.6. Enzyme-Linked Immunosorbent Assay (ELISA)

Serum levels of transforming growth factor-β1 (TGFβ1) were assayed using the RayBio^®^ Mouse TGFβ1 ELISA Kit (Catalog No. ELM-TGFβ1; RayBiotech, Norcross, GA, USA) according to the manufacturer’s instructions. Prior to assay, serum samples were diluted 1:60 in the sample diluent to ensure concentrations fell within the linear range of the standard curve. Standards, blanks, and diluted samples were added in duplicate to antibody-coated wells and incubated at room temperature. After washing to remove unbound proteins, biotinylated anti–TGFβ1 antibody was added, followed by streptavidin–HRP conjugate. Colorimetric detection was performed using the substrate solution provided, and the reaction was stopped with stop solution. Absorbance was measured at 450 nm using a microplate reader. TGFβ1 concentrations were calculated from a standard curve generated with recombinant mouse TGFβ1 provided in the kit. All samples were assayed in duplicate, and mean values of fold change relative to control were used for statistical analysis.

### 4.7. Enzyme Activity Assays

Serum antioxidant enzyme activities were measured using commercial kits according to the manufacturer’s instructions. Catalase (CAT) activity was determined with the Catalase Assay Kit (Cat. No. 707002, Cayman Chemical, Ann Arbor, MI, USA), glutathione peroxidase (GPX) activity with the Glutathione Peroxidase Assay Kit (Cat. No. 703102, Cayman Chemical), and total superoxide dismutase (SOD) activity with the Superoxide Dismutase Assay Kit (Cat. No. 706002, Cayman Chemical). For each assay, serum samples were thawed on ice, clarified by brief centrifugation, and diluted appropriately (1:5 for CAT and SOD and 1.2 for GPX) in assay buffer to fall within the linear range of the standard curve. CAT activity was quantified based on the peroxidic reaction of catalase with methanol in the presence of hydrogen peroxide, producing formaldehyde. Formaldehyde was detected calorimetrically at 540 nm using Purpled reagent. GPX activity was determined indirectly through a coupled reaction with glutathione reductase (GR). Oxidized glutathione (GSSG), generated during the reduction of cumene hydroperoxide, was recycled to reduced glutathione using NADPH. The decrease in absorbance at 340 nm, reflecting NADPH consumption, was proportional to GPX activity. Total SOD activity was measured by monitoring the dismutation of superoxide radicals generated by xanthine oxidase and hypoxanthine. The inhibition of superoxide-mediated reduction of a tetrazolium salt was detected at 450 nm. Enzyme activities were calculated from standard curves and expressed as units per milliliter (U/mL) of serum. All assays were performed in duplicate, and data are presented as mean ± standard error of the mean (SEM). Statistical comparisons between two groups were performed using unpaired Student’s *t*-tests.

### 4.8. Bioinformatics and Statistical Analysis

Functional annotation of the proteome was performed using the UniProt-GOA database (http://www.ebi.ac.uk/GOA/; accessed on 25 August 2024). Identified protein IDs were first converted to UniProt IDs and then mapped to corresponding GO terms. Proteins were classified into three categories: biological process, cellular component, and molecular function. Enrichment analysis of DEPs was carried out using Fisher’s exact test, comparing DEPs against all identified proteins. GO terms with a Benjamini–Hochberg corrected *p* < 0.05 were considered significant. GO functional hierarchies were further visualized using the R package topGO (Version 2.38.1), which generates directed acyclic graph (DAG) structures to depict parent–child relationships among enriched terms. KEGG Pathway Annotation and Enrichment Pathway annotation was conducted using the KEGG database. Enrichment analysis was performed with KOBAS 3.0, using Fisher’s exact test to assess the significance of DEP enrichment relative to the background proteome. Pathways with corrected *p* < 0.05 were defined as significantly enriched. DEPs annotated to KEGG pathways were further grouped into functional categories according to KEGG definitions. For clustering, protein expression values were log_2_-transformed, and normalized values were subjected to hierarchical clustering using Euclidean distance and average linkage. Clustering results were visualized as heat maps generated with the “heatmap.2” function in the gplots R package.

### 4.9. Analysis of SASP Protein Expression in Human Cancers

GCR-induced upregulated serum proteins (total 194) were screened as senescent cell secretory (SASP) factor using SASP atlas database (http://www.saspatlas.com; accessed on 25 August 2025) and were classified as IR-stress induced, oncogene-induced or both (Table 4). Further, to evaluate the translational relevance of 36 GCR-induced SASP proteins identified in *Apc*^Min/+^ mice, we evaluated the protein level using the Human Proteome Atlas (HPA) to identify 11 SASP factors (Table 5) whose protein expression has been reported in CRC, breast or both cancers. Finally, immunohistochemistry (IHC)-based protein expression data for 11 SASP factors were retrieved from HPA database (https://www.proteinatlas.org; accessed on 12 October 2025). The HPA contains annotated 3,3′-diaminobenzidine (DAB) chromogen with hematoxylin counterstained IHC images of histological sections from normal and cancer tissues. Manually annotated staining intensity (negative, weak, moderate, or strong), fraction of positive cells (<25%, 25–75%, >75%), and subcellular localization (nuclear, cytoplasmic, and/or membranous) data by two independent specialists following standardized guidelines were accessed and used for our analysis. As reported on the database, the final classification as high, medium, low and not detected (N.D.) is based on the combination of the staining intensity and fraction of stained cells. All analyzed data were obtained from publicly available, anonymized HPA resources in compliance with the original ethical approvals of the database.

## 5. Conclusions

In summary, this study demonstrates that exposure to full-spectrum GCR induces systemic proteomic and biochemical changes consistent with enhanced oxidative stress, senescence activation, and tumor promotion in *Apc*^Min/+^ mice. The integration of proteomic, enzymatic, and histopathological data highlights a coordinated response linking redox dysregulation and SASP activation to GCR-driven cancer susceptibility. Importantly, many of the molecular pathways implicated in this response, including oxidative damage, impaired antioxidant defenses, chronic inflammatory signaling, and senescence-associated secretory factors, are not unique to space radiation but are also engaged by IR exposures encountered on Earth, such as those arising from medical imaging, radiotherapy, occupational exposure, and environmental sources. Although the dose rate, radiation quality, and exposure patterns differ substantially between terrestrial and space settings, the overlap in affected molecular networks suggests that insights gained from GCR exposure models may inform broader understanding of radiation-induced carcinogenesis in humans. Future studies should therefore focus on delineating the temporal dynamics and dose–response relationships of these pathways, identifying causal molecular mediators, and establishing their relevance across different radiation qualities and biological contexts. Additionally, evaluating potential countermeasures, including antioxidants, senolytic agents, and anti-inflammatory interventions, may not only mitigate GCR-induced oncogenic signaling during spaceflight but also hold relevance for reducing long-term cancer risk associated with radiation exposure on Earth. Ultimately, elucidating the conserved molecular mechanisms underlying radiation-induced carcinogenesis will be critical for developing effective risk assessment strategies and protective interventions to safeguard both astronaut health during exploration-class missions and vulnerable populations exposed to ionizing radiation in terrestrial environments.

## Figures and Tables

**Table 1:** Top 25 significantly upregulated serum proteins.

Protein IDs	Protein Names	log_2_FC
A2AEP0	Odorant-binding protein 1b	1.98
P97430	Antileukoproteinase	1.68
P11591	Major urinary protein 5	1.67
Q02257	Junction plakoglobin	1.61
Q9EPU4	Cleavage and polyadenylation specificity factor subunit 1	1.57
Q8K426	Resistin-like gamma	1.53
P41245	Matrix metalloproteinase-9	1.24
Q9Z2W0	Aspartyl aminopeptidase	1.21
Q8K558	Trem-like transcript 1 protein	1.19
P01844	Ig lambda-2 chain C region	1.18
P01633	Ig kappa chain V19–17	1.00
P07724	Serum albumin	1.00
Q99JY3	GTPase IMAP family member 4	0.99
Q8BJY1	26S proteasome non-ATPase regulatory subunit 5	0.97
P57774	Pro-neuropeptide Y	0.97
Q6GQT1	Alpha-2-macroglobulin-P	0.96
P13609	Serglycin	0.94
Q8C5V8	Coiled-coil domain-containing protein 187	0.94
E9Q557	Desmoplakin	0.93
Q9D3H2	Odorant-binding protein 1a	0.91
Q9CR35	Chymotrypsinogen B	0.91
Q91 × 79	Chymotrypsin-like elastase family member 1	0.89
Q5RJG1	Nucleolar protein 10	0.89
Q00898	Alpha-1-antitrypsin 1–5	0.81
Q9Z126	Platelet factor 4	0.80

**Table 2:** Top 25 significantly downregulated serum proteins.

Protein IDs	Protein Names	log_2_FC
P54071	Isocitrate dehydrogenase [NADP], mitochondrial	−2.44
P62631	Elongation factor 1-alpha 2	−2.32
P62858	40S ribosomal protein S28	−2.14
P05367	Serum amyloid A-2 protein	−2.13
P16125	L-lactate dehydrogenase B chain	−2.06
Q99020	Heterogeneous nuclear ribonucleoprotein A/B	−2.02
P04919	Band 3 anion transport protein	−1.99
Q8VEK3	Heterogeneous nuclear ribonucleoprotein U	−1.94
P05366	Serum amyloid A-1 protein	−1.90
P01647	Ig kappa chain V-V region HP 124E1	−1.87
P06336	Ig epsilon chain C region	−1.87
Q61646	Haptoglobin	−1.82
P47915	60S ribosomal protein L29	−1.81
P54227	Stathmin	−1.79
P19123	Troponin C, slow skeletal and cardiac muscles	−1.61
Q922B1	O-acetyl-ADP-ribose deacetylase MACROD1	−1.56
P48787	Troponin I, cardiac muscle	−1.51
Q60668	Heterogeneous nuclear ribonucleoprotein D0	−1.48
P11404	Fatty acid-binding protein, heart	−1.47
Q9D7G0	Ribose-phosphate pyrophosphokinase 1	−1.44
Q61937	Nucleophosmin	−1.43
Q62167	ATP-dependent RNA helicase DDX3X	−1.43
Q9CZU6	Citrate synthase, mitochondrial	−1.43
P26443	Glutamate dehydrogenase 1, mitochondrial	−1.42
Q03265	ATP synthase subunit alpha, mitochondrial	−1.41

**Table 3:** Top 15 downregulated antioxidant enzymes in the serum of GCR exposed mice.

Protein IDs	Protein	log_2_FC
P99029	Peroxiredoxin-5, mitochondrial	−1.09
P20108	Thioredoxin-dependent peroxide reductase, mitochondrial	−0.62
P24270	Catalase	−0.84
Q9JHC0	Glutathione peroxidase 2	−0.27
P30115	Glutathione S-transferase A3	−1.37
P10649	Glutathione S-transferase Mu 1	−1.04
P15626	Glutathione S-transferase Mu 2	−0.82
O09131	Glutathione S-transferase omega-1	−1.01
P19157	Glutathione S-transferase P 1	−0.97
P35700	Peroxiredoxin-1	−0.98
Q61171	Peroxiredoxin-2	−0.79
O08807	Peroxiredoxin-4	−0.32
O08709	Peroxiredoxin-6	−0.65
P08228	Superoxide dismutase [Cu-Zn]	−0.73
P10639	Thioredoxin	−0.45

**Table 4:** Classification of 36 GCR-induced SASP factors based on the SASP Atlas.

Protein (Symbol)	IR-Induced	Oncogene-Induced
Carboxypeptidase Q (CPQ)	Yes	--
Cleavage and polyadenylation specificity factor subunit 1 (CPSF1)	Yes	--
Platelet glycoprotein V (GP5)	Yes	--
Phosphatidylinositol-glycan-specific phospholipase D (GPLD1)	Yes	--
Transforming growth factor beta-1 (TGFβ1)	Yes	--
Collagen alpha-1(XVIII) chain (COL18A1)	Yes	--
Retinol-binding protein 4 (RBP4)	Yes	--
Xaa-Pro dipeptidase (PEPD)	Yes	--
Beta-2-microglobulin (B2M)	--	Yes
Lysosomal alpha-mannosidase (MAN2B1)	--	Yes
Integrin beta-1 (ITGB1)	--	Yes
Integrin beta-3 (ITGB3)	--	Yes
Receptor-type tyrosine-protein phosphatase kappa (PTPRK)	--	Yes
Matrix metalloproteinase-9 (MMP9)	--	Yes
Tetranectin (CLEC3B)	--	Yes
Insulin-like growth factor-binding protein 6 (IGFBP6)	--	Yes
Histone H4 (HIS1H4A)	--	Yes
Apolipoprotein A-1 (APOA1)	--	Yes
Thrombospondin-2 (THBS2)	--	Yes
Alpha-1B-glycoprotein (A1BG)	--	Yes
Collagen alpha-2(V) chain (COL5A2)	--	Yes
Endoplasmic reticulum resident protein 44 (ERP44)	--	Yes
Cystatin-C (CST3)	common	
Metalloproteinase inhibitor 2 (TIMP2)	common	
Beta-hexosaminidase subunit alpha (HEXA)	common	
72 kDa type IV collagenase (MMP2)	common	
Thrombospondin-1 (THBS1)	common	
Serum albumin (ALB)	common	
Insulin-like growth factor-binding protein 2 (IFGBP2)	common	
Insulin-like growth factor-binding protein 4 (IGFBP4)	common	
Insulin-like growth factor-binding protein 5 (IGFBP5)	common	
Prosaposin (PSAP)	common	
Neutral alpha-glucosidase AB (GANAB)	common	
Filamin-A (FLNA)	common	
Myeloid-derived growth factor (MYDGF)	common	
Proteasome subunit alpha type-6 (PSMA6)	common	

**Table 5:** GCR-induced serum SASP protein status in human colon and breast cancer tissue.

SASP Factor	Breast (% Tumors)				Colorectal (% Tumors)			
	High	Medium	Low	ND *	High	Medium	Low	ND *
Serum albumin (ALB)	18.2	45.5	27.3	9	0	66.7	25	8.3
Cystatin-C (CST3)	33.3	33.3	16.7	16.7	50	50	0	0
Metalloproteinase inhibitor 2 (TIMP2)	0	70	30	0	0	50	41.7	8.3
Thrombospondin-1 (THBS1)	66.7	8.3	16.7	8.3	58.3	25	16.7	0
Insulin-like growth factor-binding protein 6 (IGFBP6)	0	9.1	45.4	45.5	18.2	45.4	27.3	9.1
Histone H4 (HIS1H4A)	25	75	0	0	72.7	27.3	0	0
Xaa-Pro dipeptidase (PEPD)	0	75	16.7	8.3	25	75	0	0
Prosaposin (PSAP)	100	0	0	0	100	0	0	0
Neutral alpha-glucosidase AB (GANAB)	0	81.8	18.2	0	16.7	83.3	0	0
Endoplasmic reticulum resident protein 44 (ERP44)	0	100	0	0	75	25	0	0
Proteasome subunit alpha type-6 (PSMA6)	16.7	83.3	0	0	45.4	45.5	9.1	0

High > 75%, Medium 25–75% and low < 25%. * ND = Not detected.

## Data Availability

The original contributions presented in this study are included in the article/Supplementary Material. Further inquiries can be directed to the corresponding author. The raw data supporting the conclusions of this article will be made available by the authors on request.

## Supplementary Materials

The following supporting information can be downloaded at: https://www.mdpi.com/article/10.3390/ijms27010211/s1.

## Abbreviations

The following abbreviations are used in this manuscript:

Apc	Adenomatous Polyposis Coli Gene
CAT	Catalase
cGy	Centigray
CRC	Colorectal Cancer
DAG	Directed Acyclic Graph
DEPs	Differentially Expressed Proteins
GCR	Galactic Cosmic Radiation
GI	Gastrointestinal
GO	Gene Ontology
GPX	Glutathione Peroxidase
HZE ions	High-Charge, High-Energy Ions
IR	Ionizing Radiation
KEGG	Kyoto Encyclopedia of Genes and Genomes
LET	Linear Energy Transfer
MMP9	Matrix Metalloproteinase 9
MMPs	Matrix Metalloproteinases
RBE	Relative Biological Effectiveness
SASP	Senescence-Associated Secretory Phenotype
SOD	Superoxide Dismutase
SPEs	Solar Particle Events
TGF1	Transforming Growth Factor Beta-1
Z	Atomic Number
