# Supplementary material for: Elevated SASP Factors, Reduced Antioxidant Enzymes, and Increased Tumor Susceptibility in Space Radiation-Exposed ApcMin/+ Mice
Source: Int J Mol Sci. 2025 Dec 24;27(1):211. doi: 10.3390/ijms27010211 (PMC12785459; doi:10.3390/ijms27010211)
Supplement: Supplementary file 1 [file ijms-27-00211-s001.zip › Figure S1.pdf]

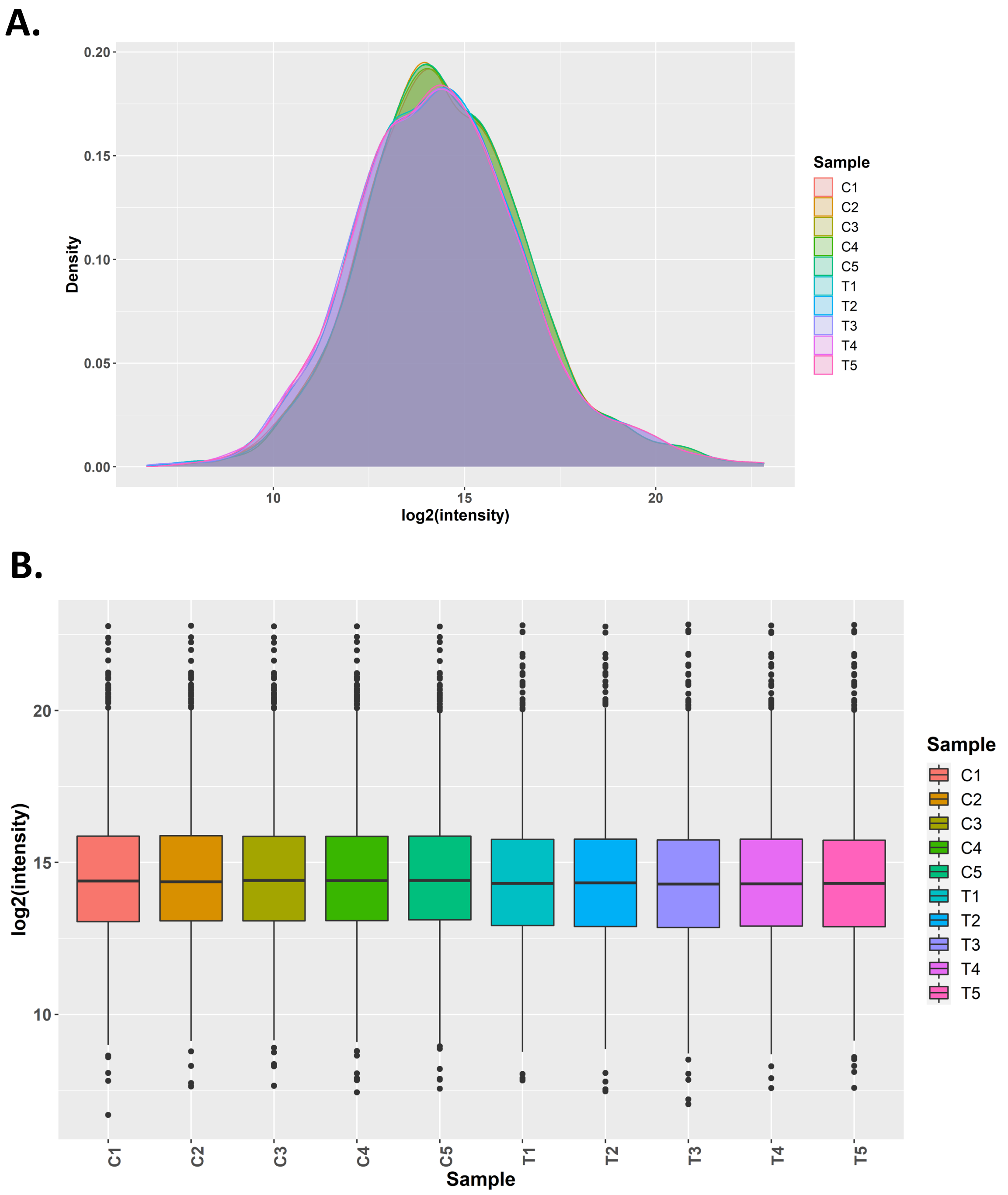

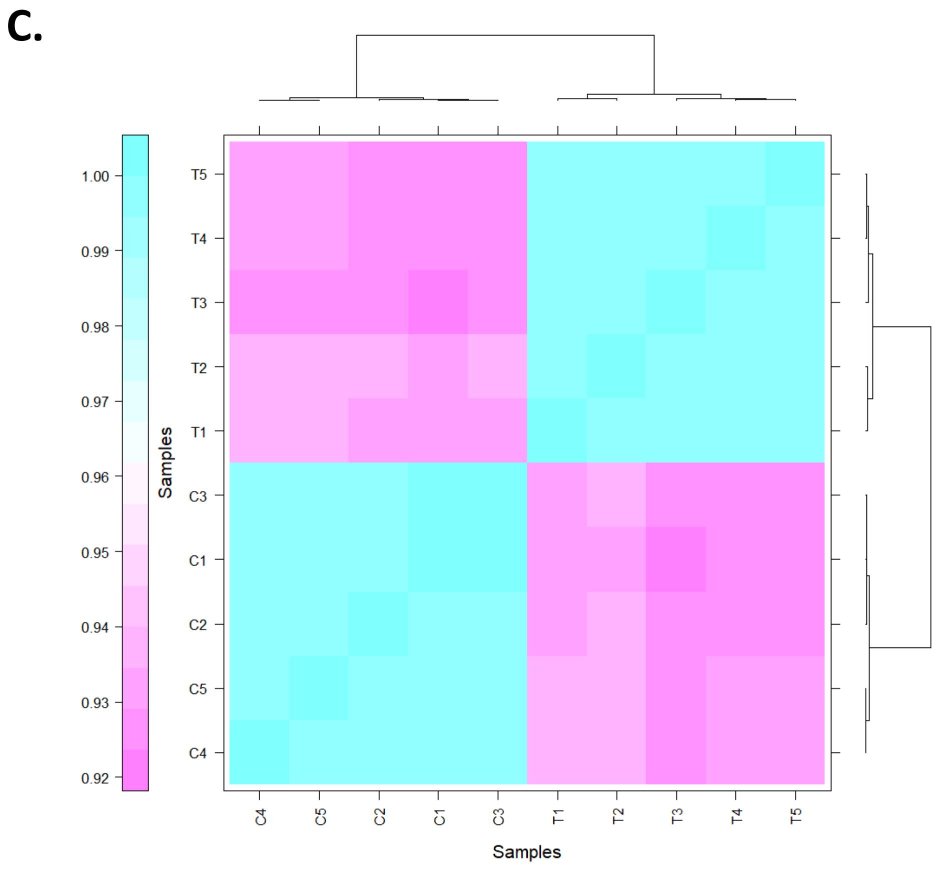

Figure S1. Quality control assessment of TMT-based serum proteomics data
(A) Density distribution of log2-transformed protein intensities across all samples (C1–C5, controls; T1–T5, GCR-treated). Overlaid kernel density curves show comparable global intensity distributions among samples, indicating minimal technical variability prior to downstream analysis. (B) Boxplots of normalized log2 intensities for each sample demonstrate consistent median intensity levels and similar distribution ranges across all control (C1–C5) and GCRsim (T1–T5) channels, confirming effective normalization of TMT reporter ion abundances. (C) Sample–sample correlation heatmap with hierarchical clustering computed from normalized protein intensities. The clustering pattern reveals clear separation between control and GCRsim samples, reflecting underlying biological differences and high within-group similarity.
